# Increasing Coverage in Mass Drug Administration for Lymphatic Filariasis Elimination in an Urban Setting: a Study of Malindi Town, Kenya

**DOI:** 10.1371/journal.pone.0083413

**Published:** 2014-01-14

**Authors:** Doris W. Njomo, Dunstan A. Mukoko, Nipher K. Nyamongo, Joan Karanja

**Affiliations:** 1 Eastern and Southern Africa Centre of International Parasite Control (ESACIPAC) Kenya Medical Research Institute (KEMRI), Nairobi, Kenya; 2 Division of Vector Borne and Neglected Tropical Diseases, Ministry of Health, Nairobi, Kenya; 3 Malindi District Hospital, Ministry of Health, Malindi, Kenya; Johns Hopkins University, United States of America

## Abstract

**Introduction:**

Implementation of Mass Drug Administration (MDA) in urban settings is an obstacle to Lymphatic Filariasis (LF) elimination. No urban-specific guidelines on MDA in urban areas exist. Malindi district urban area had received 4 MDA rounds by the time the current study was implemented. Programme data showed average treatment coverage of 28.4% (2011 MDA), far below recommended minimum of 65–80%.

**Methods:**

To identify, design and test strategies for increased treatment coverage in urban areas, a quasi-experimental study was conducted in Malindi urban area. Three sub-locations with lowest treatment coverage in 2011 MDA were purposively selected. In the pre-test phase, 947 household heads sampled using systematic random method were interviewed for quantitative data. For qualitative data, 12 Focus Group Discussions (FGDs) with single sex adult and youth male and female groups and 3 with community drug distributors (CDDs) were conducted. Forty in-depth interviews with opinion leaders and self-administered questionnaires with District Public Health officers purposively selected were carried out. The quantitative data were analyzed using SPSS version 16 and statistical significance assessed by χ^2^ test.The qualitative data were analyzed manually according to study's themes.

**Results and Discussion:**

The identified strategies were implemented prior to and during 2012 MDA in two sub-locations (experimental) while in the third (control), usual MDA strategies were applied. In the post-test phase, 2012 MDA coverage in experimental and control sub-locations was comparatively assessed for effect of the newly designed strategies on urban MDA. Results indicated improved treatment coverage in experimental sub-locations, 77.1% in Shella and 66.0% in Barani. Central (control) sub-location also attained high coverage, 70.4% indicating average treatment coverage of 71%.

**Conclusion:**

The identified strategies contributed to increased treatment coverage in experimental sites and should be applied in urban areas. Due to closeness of sites, spillover effects may have contributed to increased coverage in the control site.

## Introduction

Lymphatic filariasis (LF) also known as ‘elephantiasis’ caused by filarial worms and transmitted by mosquitoes is ranked as the second largest cause of disability in the world [1]. Over a billion people live in areas where they are at risk of infection due to continuous exposure to infected mosquito vectors [2]. It is “a disease of poverty” which affects poor people living in poor areas often with limited access to safe water and sanitation facilities. Lymphatic filariasis is a painful and disfiguring disease, which undermines health, economic opportunities and social interaction. Infection leads to a variety of clinical manifestations, including lymphoedema of the limbs and the genitalia (especially hydrocele). About 41 million people worldwide have visible signs, a further 76 million have hidden infections, most often with microfilariae in their blood and hidden internal damage to their lymphatic and renal systems and about 44 million infected patients have recurrent infections and abnormalities of renal functions [3], [4]. In sub-Saharan Africa, it is estimated that about 512 million people are at risk of the infection and about 28 million are already infected. Of this number, there are 4.6 million cases of lymphoedema and over 10 million cases of hydrocele. These represent about 40% of the global burden of the disease [5]. In Kenya, LF affects 3.5 million people living in the coastal area and villages along the River Sabaki in Malindi had an overall prevalence of microfilaraemia of at least 7.1% after the first 2 MDA rounds [6].

Lymphatic filariasis has been identified as a potentially eradicable disease by the International Task Force for Disease Eradication [7]. The recognition that two-drug single dose treatment strategies (albendazole and ivermectin or diethylcarbamazine citrate, DEC) are significantly more effective than treatment with either drug alone, has been a major advancement in the development of control regimens for lymphatic filariasis [8], [9], [10]. The principal objective of the Global Programme to Eliminate Lymphatic Filariasis (GPELF) is to interrupt transmission of infection by decreasing the parasite population in human hosts through annual MDA of single-dose DEC or ivermectin in combination with albendazole [11]. Interrupting the transmission of the parasites that cause the disease requires careful identification of the endemic areas as well as the use of drugs designed to reduce microfilaraemia and transmission intensity [12]. For elimination to occur, 80% of the eligible- at risk populations have to comply with MDA annually for 4–6 rounds [13]. A number of studies have underscored the importance of compliance in the elimination programmes [14]. By the end of 2009, 2.8 billion doses of medicine had been administered to a cumulative targeted population of 845 million individuals in 53 of the 81 endemic countries with 29 of the 53 countries achieving full geographical coverage and 20 having already completed five or more rounds of MDA in all endemic areas [15]. The technical advisory group has noted that drug delivery to people who do not consume them has an adverse effect on the programme impact. For this reason, it encourages programme managers to implement their programme using the principal of directly-observed treatment [16]. Achieving high treatment coverage in urban settings has been reported as a challenge in studies conducted in Colombo 53.4% [17] and Orissa 42% [18].

In Kenya MDA for LF elimination was first implemented in 2002 in Kilifi District and in 2003 scaled up to include Kwale and Malindi Districts. Prior to the implementation of the current study, Malindi urban area had received four MDA rounds and the programme data showed that the treatment coverage achieved was far below (48%, 46%, 46.5% and 30%) the recommended 80% of the eligible population. Community volunteers, known as community drug distributors (CDDs) selected by the community members to deliver drugs to individuals at their homes were used in the four rounds of treatment. Each CDD was expected to cover a total of 250 households. The District Medical Officer and political authorities were first to be sensitized on MDA (endemicity of the area, purpose of mass treatment, drugs used, method of distribution, length of distribution and role of WHO in the programme) followed by peripheral health providers who then sensitized community leaders. The community leaders through open meetings at community level sensitized community members and together they selected CDDs. The CDD selection criteria include: ability to read and write; keep records; trustworthiness; well known in the community members; and willingness to distribute drugs to all eligible persons in allocated areas without remuneration by the project [19]. The CDDs were trained by health personnel and the community was at liberty to decide the best way to incentivise them. The distribution of drugs was done house-to-house and the whole exercise would take a single day while on the following day, revisits would be conducted to those missed out on the initial day. During the distribution exercise, the health personnel were on standby to manage side effects,usually minor and included nausea, headache, dizziness, fever, malaise, decreased appetite and vomiting.

The global LF elimination campaign is faced with the challenge of persuading people who have no symptoms of the disease to take the drugs [4]. It is important to have no group of persons remaining totally untreated because such a group if infected forms a reservoir of microfilariae (mf) contributing to continued transmission of infection [14].

Achievement of high treatment coverage is a key element in the elimination of LF. The results of a study conducted in the rural areas of Malindi District in 2009 indicate that where the populations had higher social status, treatment coverage achieved was low and a dislike for the current drug distribution method due to mistrust of the distributors was a common reason for the low coverage [20], [21]. Implementing successful MDAs in the urban areas is characterized by challenges such as: population registration prior to MDA due to the presence of non-resident populations; limited accessibility of the urban dwellers to receive door-to-door treatment and necessity to acquire specific parental consent. Inadequate programme support and advocacy for effective communication strategy; high LF awareness but low risk perception; low compliance due to insufficient information; education and communication materials; lack of uniformity in need for MDA across different socio-economic strata and CDDs inadequacy and demand for higher incentives are other challenges of successful MDAs in urban areas. The current study sought to identify, design and test strategies that could be used to developguidelines for achieving high treatment coverage in an urban setting and to identify possible pitfalls that could be a hindrance to achieving high treatment coverage in such urban settings.

## Methods

### Ethics statement

Ethical clearance was received from the Kenya Medical Research Institute (KEMRI)/National Ethical Review Board (Protocol Number 1988) and written informed consent sought from all the study participants. All the participants were adults above the age of 18 years and therefore no parents/guardians were expected to give consent on behalf of a minor for participation in the study.

### Study area

Malindi District is located 120 kilometers northeast of Mombasa, and lies between latitudes 2.2° and 4° south and between longitudes 39° and 41° east. It covers a geographical area of 7,605 km^2^ with a total population of 384,643 [22]. The District is endemic for LF caused by *Wuchereria bancrofti* and studies conducted in villages along the River Sabaki in Malindi showed a filarial endemicity of at least 7.1% [6]. Malindi District has 3 hospitals (1 government and 2 private); 24 dispensaries (17 government and 7 NGO) and 4 private chemists. The average distance to the nearest health facility for urban areas is 1 km and 3 kms for rural areas. Most of the health facilities are therefore not generally accessible to the majority of the population. High poverty levels, cost sharing and long distances inhibit people from visiting these facilities. The doctor/patient ratio is 1∶19,502. The most prevalent diseases are; malaria, respiratory diseases, diarrhea, intestinal worms, STIs, anemia and eye infections. The utilization of health facility for child delivery is at 41% and reasons for low usage are distance and low socio-economic status [23].

**Figure 1:**
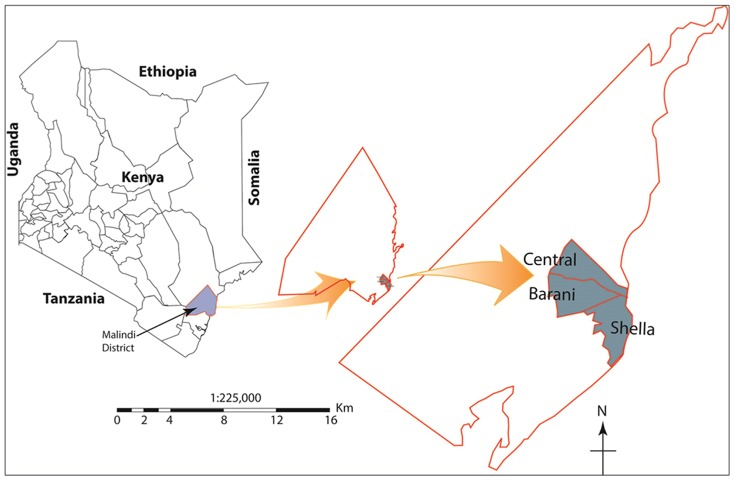
Map of Kenya showing Malindi District and urban study area of Central, Barani and Shella.

Malindi Urban area is a town on Malindi Bay at the mouth of the Sabaki River, lying on the Indian Ocean coast of Kenya. The population of Malindi Town is 123,965 [22] and it is the capital of the Malindi District.Tourism is the major industry and the city is exceptionally popular among Italian tourists. Malindi is served with a domestic airport and a highway between Mombasa and Lamu (Figure 1).

### Study design and setting

This was a quasi-experimental study which utilized both quantitative and qualitative methods. Based on the May 2011 MDA Programme data, three of the five sub-locations of Malindi Urban area were purposively selected for the study as they had achieved the low (Barani, 26.7%, Central, 32% and Shella 26.5%) treatment coverage compared with the other 2 sub-locations (Kijiwetanga, 69.4% and Sabaki, 72.3%). In the pre-test phase, the May 2011 MDA was used as a basis of enquiry on the usual MDA process and to identify and design new strategies aimed at increasing treatment coverage. The newly designed strategies were then tested in selected experimental sub-locations (Barani and Shella) prior to and during the October 2012 MDA and thereafter an impact assessment was conducted to compare differences/similarities in treatment coverage between the experimental sub-locations with newly designed strategies and the control sub-location (Central) with the usual MDA strategies.

### Study population

A total of 947 households were selected through systematic random sampling and interviewer-based questionnaires administered to the heads or adult representatives of the three sub-locations by trained research assistants for quantitative data. For qualitative data, in-depth interviews were conducted with 40 opinion leaders purposively selected based on their being leaders of social, political and religious groupings. To elicit more information on opportunities for and barriers towards MDA, twelve focus group discussions (FGDs) were carried out with adult and youth male and female single-sex groups and 3 with CDDs who had distributed drugs during the previous MDA round and moderated by the lead author assisted by trained field assistants using *Kigiriama* and *Kiduruma*, the local languages. Notes were taken during the FGDs and audiocassettes used to tape record all the information in the local languages. The tapes were later transcribed and translated into English.The hard copies of both the qualitative and quantitative data were stored in lockable and secure cabinets. To ensure quality control, the soft copies were stored in computers with passwords, with authorized access by the PI to the study investigators.

### Statistical analysis

The quantitative data were analyzed using SPSS version 16.The responses to open-ended questions were coded before entry. Equivalent responses were pooled to arrange the responses in different categories. Two-way tables were used to compare categorical data and the statistical significance of differences in coverage was assessed by the χ^2^ test. A *P* value of ≤0.05 was considered statistically significant. Proxy measures such as ownership of property were used to categorize the households into three socio-economic strata. The quantitative data was collected before the qualitative data. This was mainly to generate meaning for the various patterns observed from the preliminary quantitative data analysis.

The qualitative data from various sources were analyzed manually according to the themes of the study and triangulated for cross verification. The triangulation helped increase the credibility and validity of the results by continuously cross-checking the data from the various sources.The data were examined separately for each of the three socio-economic strata. Similar questions were asked to various types of respondents and data were triangulated to check for consistency and divergence of views.The study's dependent variable was treatment coverage levels and the independent variables whose outcomes were either binary or categorical included: client-related factors (knowledge and perceptions); provider-related factors (selection, training, drug supplies, remuneration, distribution method and duration) and programme sustainability factors (stakeholders, social structures and channels).

### Intervention activities

The study was conducted between July 2011 and October 2012 and a review of the existing literature on coverage factors in MDA programmes was used to identify, design and test existing strategies/opportunities for increased MDA coverage and to identify barriers hindering high MDA coverage. The existing strategies/opportunities identified, designed and tested were; combination of awareness creation methods and materials including repeated announcement using public address system, dissemination of adequate MDA information by use of a brochure explaining LF transmission and need for MDA using local language; engagement of Malindi district urban area stakeholders in the entire MDA process and the use of the door-to-door method of drug distribution. The barriers identified were: limited number of CDDs to cover all households in the urban area, inadequate training of CDDs and supervisors, limited duration of drug distribution, limited incentives for health workers, supervisors and CDDs, lack of identification badges for CDDs and of DOT.

## Results

### Background characteristics of the study participants

The mean age of the household heads or representatives majority (55.2%) of whom were female was 35.45 years (SD = 11.85, median 33.0 and range 18–89 years). A large proportion, 67.8% was in marital unions, 21.7% was single, 6% divorced and 4.4% was either widow or widower. Slightly more than one-half (51.1%) of the respondents were Christians, 41.9% Muslims while 6.8% were non-practicing. Regarding literacy levels, slightly more than three-fifths, (61.1%) of the household heads were literate, had at least completed primary and/or gone for higher education while 38.9% were illiterate; no formal schooling or incomplete primary education. With regard to main occupation, slightly more than two-fifths, 41.2% of the households were businessmen/businesswomen, 13.7%, casual laborers, 13.5%, salaried workers and 16.8%, housewives.

The household heads were drawn from three sub-locations of the study area, Table 1.

**Table 1:** Number of Household Heads by Sub-location.

Sub-location	No. of Households	%
Barani	316	33.4
Central	314	33.2
Shella	317	33.5
Total	947	100

Slightly more than four-fifths (82.5%) of the opinion leaders was male and their age range was 25–67 years. One quarter was community residence leaders,20% social group leaders another one quarter was in employment (doctor, nurse, teacher etc.) while one-eighth was preachers. A high proportion, 60% of the opinion leaders was Christians, 37.5% Muslim and the remaining was non-practicing. A large majority (70%) of the opinion leaders had attained secondary school or further education, 17.5% had primary school education while 7.5% had never gone to school. The remaining 5% of the participants had undergone informal schooling.

The single-sex adult and youth male and female FGDs participants included adults (35 years and above) and youth (18 to 34 years) respondents of homogenous characteristics. The CDDs who participated in the separate FGDs included both male and female drug distributers who had distributed anti-filarial drugs in the 3 sub-locations during May 2011 MDA. Each FGD contained a minimum of 8 and a maximum of 12 participants and standard procedures [24] were adhered to.

### Reasons for not taking Drugs

**Figure 2:**
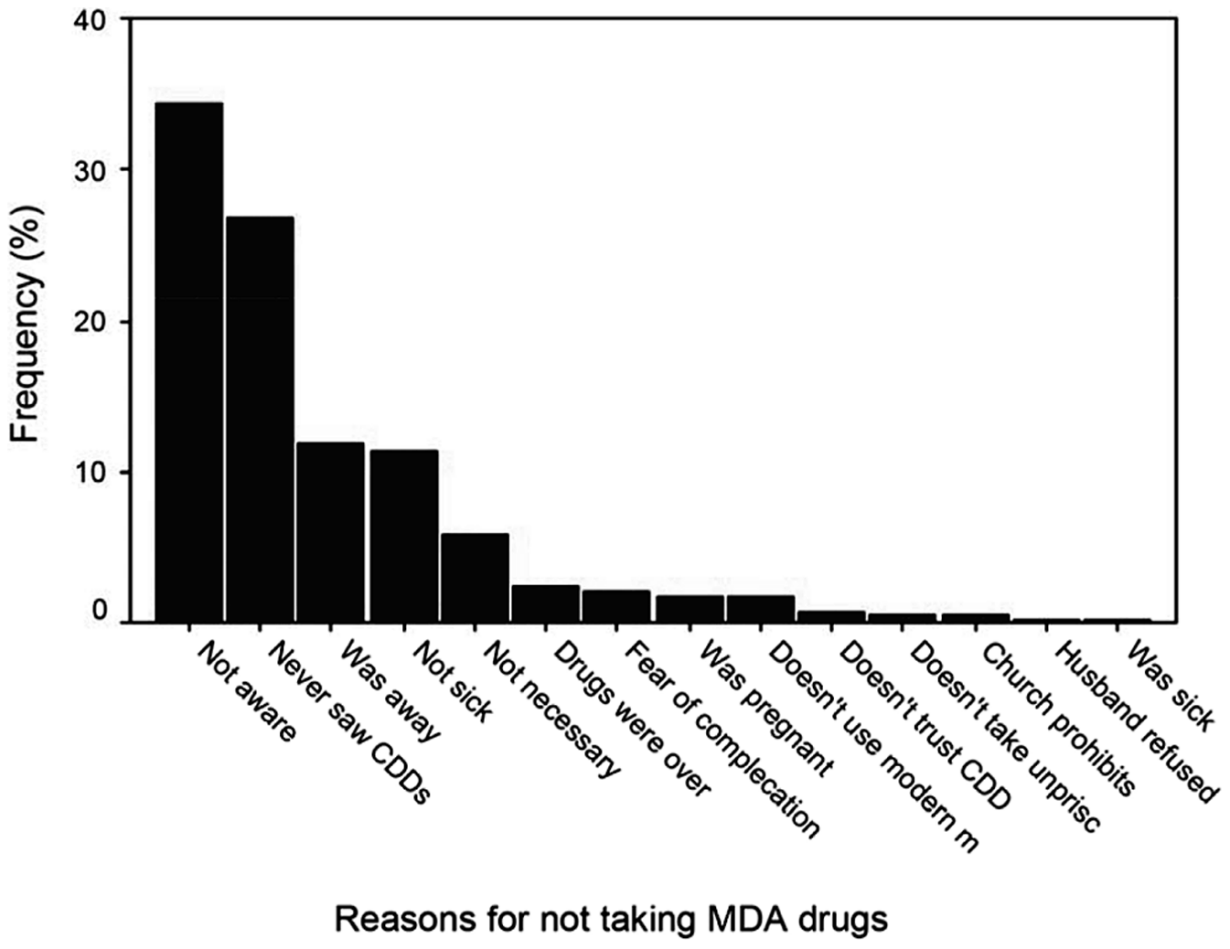
Reasons for not taking drugs during MDA.

The reasons for not taking drugs among those who did not were significantly different among household heads in the 3 socio-economic strata P<0.01. Not being aware of the MDA and CDDs not visiting the household were the most prominent reasons given for not taking drugs during the 2011 MDA. Among the respondents in the high, middle and low socio-economic strata,45.7%, 34% and 31% respectively were not aware of the MDA and therefore did not take the drugs. The CDDs not visiting the household to administer drugs was another reason given by 20%, 29% and 24% of the respondents in the high, middle and low socio-economic strata respectively (Figure 2).

The opinion leaders further commented on the barriers hindering their community members' participation in the MDA. Lack of awareness and inadequate information about the MDA was the leading barrier reported by one-half of the opinion leaders while issues to do with CDDs such as poor interaction, language barrier and limited numbers were indicated by slightly less than one-fifth of the respondents.

A 42 year old male teacher (opinion leader) from Barani Sub-location stated:

“*I wish next time they conduct a distribution like this they at least set aside sometime to educate the people first so that they understand what is happening.Unlike in the past MDA the drugs came immediately after the information had been given.*”

A 28 year old male Rehabilitation Centre representative (opinion leader) from Shella Sub-location further indicated that:

*“The community members require more frequent information to be educated and know well about the LF disease and how to prevent it.”*

A CDD from the Shella sub-location FGD also stated that:

*“They should announce and also sensitize people like for a week so that everyone is aware of what is being done around the community.”*

### Source of Information about MDA

**Figure 3:**
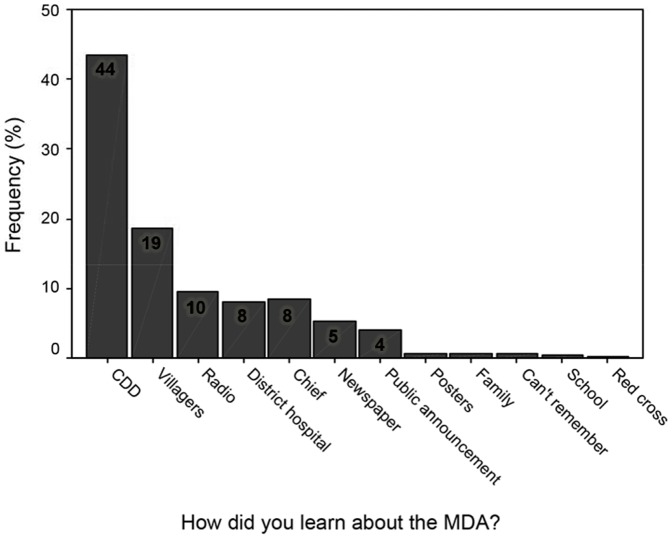
Sources of Information on MDA.

The source of information about MDA was significantly different in the 3 socio-economic strata- high, middle, low- P<0.05, 0.01 and 0.01 respectively. The CDDs were the most prominent source of information; (42%, 42% and 47% in high, middle and low socio-economic strata respectively), followed by villagers (16%, 22% and 13% respectively), the District hospital (9%, 7% and 9% respectively) and radio, at 9%, 10% and 9% respectively in the high, middle and low socio-economic strata (Figure 3).

With regard to the kind of facilitation given to the community members during MDA, less than one-fifth of the opinion leaders indicated that no facilitation was given while one- half indicated that the village leaders, community drug distributors and other community members facilitated the MDA through awareness creation. Media through radio and television was indicated as an effective mode of awareness creation by about two-thirds (26) of the opinion leaders,

Regarding the improvement of awareness creation on MDA, one-third of the opinion leaders mentioned the use of public gatherings and chief's meetings (*barazas*) for informing the community members about the drug distribution. Churches, mosques and other religious institutions as effective forums for creating awareness were also indicated by one-third of the opinion leaders. The need to use a combination of health promotion and awareness creation materials (posters, town announcers, banners, road shows and local artists) for increased awareness were expressed by three-fifths of the opinion leaders. One-fifth of the opinion leaders also indicated that the use of the Provincial Administration and Medical Officer of Health would be effective for increased awareness creation on drug distribution.

A 46 year old male village elder from Central Sub-location stated that:

*“The Provincial Administration should be involved as people take them more seriously. The village elders should also be used in creating awareness. About the misconceptions people have, the household heads are the ones who should convince their members to take the medication.”*

A large majority of the participants in all FGDs emphasized the need to adequately create awareness and further indicated the importance of using all modes of awareness creation so as to increase coverage. Among the most expressed preferred modes of awareness creation by a large majority of the participants in all FGDs included posters, churches, mosques, schools, local radio stations, road shows, newspaper, drama, District Commissioners, District Officers, theatre groups, loudspeakers and chief's meetings (*barazas*).

One participant in the adult male FGD held in Barani Sub-location stated that: “*Awareness creation should be through churches and mosques because very many people attend these places so that the information can reach a lot of people within a short time.*”

The CDD FGDs participants were also asked to give an account of the difficulties they experienced during the MDA. Among the difficulties experienced by a large majority of the CDDs included inadequate community awareness of the MDA.

A CDD in the FGD conducted in Shella Sub-location further stated that:

*“Some community members were not aware so we took long hours because we had to explain to them in detail before giving them the drugs and also to convince them.”*

### Type of Information Received

The type of information received about MDA also varied, but prominently for prevention of LF, at 59%, 76% and 71% respectively in high, middle and low socio-economic strata and mass drug distribution against LF at 10%, 5% and 6% respectively in high, middle and low socio-economic strata. Opinion on the information source was also significantly different in the 3 socio-economic strata, P<0.001. A higher proportion (36% and 30%) of the respondents respectively in middle and low socio-economic strata reported that they perceived the type of information they received as reliable against 19% of the respondents in the high socio-economic strata who were of the same opinion.

With regard to Information, Education and Communication (IEC) materials, a large majority,(four-fifths) of the CDDs in all the FGDs, indicated that materials should be done using the local language and frequent reminders should be made by the town announcers to ensure that all community members are made aware of the MDA. Majority of the CDDs however expressed that they felt that the posters had adequate information enhanced by the photographs and pictures and served the purpose well.

A CDD in Shella Sub-location FGD stated:

*“The awareness creation materials should add more details on causes of the disease e.g. draw a mosquito to show that that's what causes the disease.”*

A CDD in the Central Sub-location FGD stated:

*“The pictures on the leaflets are convincing because they shock people hence making them to take drugs.”*

### Opinion on Drug Distribution Method

Opinion on whether the drugs should be distributed by the same method (house-to-house) as in the 2011 MDA in subsequent rounds, was significantly different in the 3 socio-economic strata, P<0.001; less than 45% of respondents in the high socio-economic strata compared to 71% and 61% respectively in the middle and low strata were of the opinion that the drugs be distributed using the house-to-house method.

### Preferred Method of Drug Distribution

**Figure 4:**
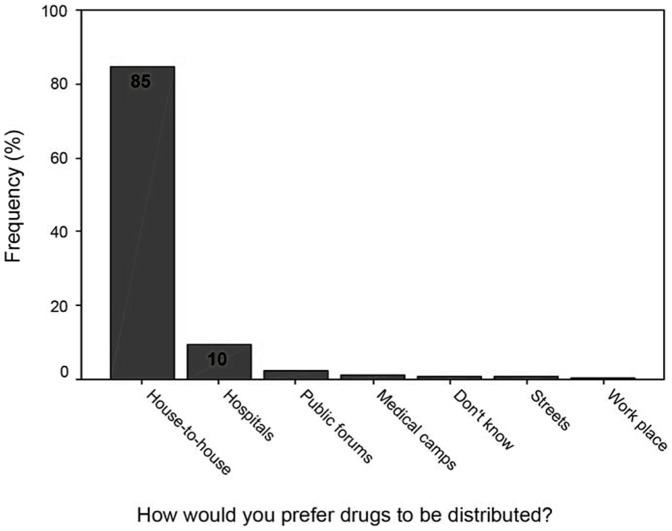
Preferred Method of Drug Distribution.

The preferred way for drug distribution significantly differed in the 3 socio-economic strata, P<0.05.The house-to-house was the most preferred method of distribution, at 80%, 86% and 83% respectively in the high, middle and low socio-economic strata and distribution through hospitals (health facilities), at 14%, 10% and 8% of respondents in high, middle and low socio-economic strata respectively (Figure 4).

A large majority of the participants in all the FGDs mentioned that they were satisfied with the house-to-house method of drug distribution as many people could be reached and all those who should take the drugs would not be missed.

One participant in the female youth FGD in Shella sub-location stated that: *“The system of house-to-house is the best because parents know their children's age so it is easy to separate those who are not supposed to take drugs other than the system of putting the drugs in one public place where everybody comes and picks the drugs you will find that even those who are not eligible take the drugs.”*

A large majority of the opinion leaders indicated that the door-to-door method of drug distribution is good as many people can be reached in their homes and all household members can take drugs together.

A 60 year old male Village Elder (opinion leader) from Barani sub-location further stated that: “*This method of distributing drugs door-to-door is just good because each and every household will be visited and the choice of taking drugs will remain to be of the household head and even the community members want it that way because they think that is what is best.*”

A large majority of the participants in the 3 CDD FGDs further emphasized that the door-to-door method of drug distribution is good but felt that coverage for all households was not achieved due to time limitations since the duration of MDA was too short and the distances to be covered were vast. In one of the FGDs, majority of the participants indicated that those community members who were at work during the day were not reached for MDA.

One participant in the Shella sub-location CDDs FGD indicated that: “*I like the door-to-door method of drug distribution and I would participate for as long as I can because I am interested in helping my community members*”.

A CDD in the Barani FGD further indicated that: *The number of CDDs should be increased and the duration of distribution extended.*”

A large majority (four-fifths) of the CDDs in all FGDs gave suggestions of ways of improving the Programme and these included educating the community members and creating adequate awareness well in advance, increasing the number of CDDs as well as providing them with adequate training.

A CDD in Barani Sub-location FGD further indicated that:*“The CDDs should be well trained and their identities should be made. The CDDs should be well taken care of e.g. we need transport allowance, badges, T-shirts and lunch.”*

The following programme sustainability strategies were identified and implemented in the study sites:

#### A one day meeting of all stakeholders of Malindi Urban area

A one day meeting with all stakeholders of Malindi urban area was held in order to plan for the MDA. The Stakeholders included local leaders i.e. village elders, chiefs and assistant chiefs, religious leaders i.e. imams and Christian leaders, leaders of women and men groups, local administration, community health workers and business representatives from each of the three sub-locations. The Hosts of the meeting were the DPHO, DHEO and the Study Team.This was done to ensure an all-inclusive planning of the MDA process. From the meeting, a comprehensive plan and timeframe for the MDA process was made, duties and action points allocated to specific stakeholders; and resources (from community, MoH and study team) for effective MDA distributed among the three sub-locations.

Based on the population of each sub-location, the following was agreed upon: Shella had 14 stakeholder representatives and was required to select 2 supervisors and 50 CDDs. Barani had 4 stakeholder representatives and was required to select 1 supervisor and 30 CDDs. Central had 3 stakeholder representatives and was required to select 1 supervisor and 20 CDDs. It was then agreed upon that the stakeholders would contact the various village elders who would then help them in CDD selection. The remaining supervisors were health workers and hence were selected from the hospital.

#### Drug distribution method

The most preferred (75% of the household heads) method of drug distribution i.e. door-to-door was identified for use in 2012 MDA. Only 25% indicated that the distribution should be done by use of churches, mosques, schools and public gatherings. Nearly all participants in all the FGDs expressed their satisfaction with the door-to-door method of drug distribution. The door-to-door method which had been used in the 2011 round was still the most preferred as indicated by 29 (72.5%) of all the opinion leaders from the 3 sub-locations.

#### Drug distributors

The Drug distributors were thoroughly trained, their numbers increased so that every distributor was expected to cover a maximum of 250 households within 3 days. The CDDs from the experimental sub-locations were provided with identification badges and T-Shirts to use during the exercise while those from the control sub-location only received badges while T-Shirts were given after the distribution exercise. All distributors were supervised and given a transport allowance. The duration of drug distribution was 3 days, one day for household registration and inter-personal communication using a leaflet that has been used previously and which the community members expressed having satisfaction with. The leaflet with information on LF and MDA was provided to each household. The second day was for drug distribution and the third for follow up for those missed on the distribution day. Emphasis on the need to observe drug swallowing was impressed upon the CDDs.

#### Awareness Creation and Community Mobilization

This was done by use of posters which were posted in public places, shopping centers, market places, schools, hospitals, churches, mosques and all other appropriate places. Announcements on the mass campaign were done at the Churches and Mosques and Schools. Street banners were also pasted in shopping Centres and market places. The number of posters, street banners and announcements in Churches and Mosques was greater in the experimental sub-locations compared to the control sub-location. Town announcement by use of a loudspeaker were made for 2 days, once before the MDA day and once during the MDA day only in the experimental sub-locations. The communities were informed about the drug distribution during Chief's meetings where CDDs selection was also conducted. The District Commissioner and District Officers also held public meetings with the community members and a health officer was invited to give information and education on LF and MDA. Local language was used in all awareness creation materials and methods.

In the post-test phase, an impact assessment of treatment coverage between experimental (sites with newly designed strategies) in comparison with control (site with the usual MDA strategies) was conducted after the October, 2012 MDA. The results showed a significant increase in treatment coverage in all three sub-locations (Table 2). The average treatment coverage for the 3 sub-locations was 71%. Shella and Barani sub-locations (experimental sites) recorded treatment coverage of 77.1% and 66.0% respectively while Central sub-location (control site) recorded treatment coverage of 70.4%.

**Table 2:** Treatment Coverage by Sub-Location (2012).

Sub-Location	Barani	Central	Shella
Total Households	4,438	1,736	6,345
Number Treated	18,093	7,675	26,994
Total registered	27,432	10,902	35,023
**% treated**	**66.0%**	**70.4%**	**77.1%**

### Study Limitations

The current study's main limitation was inability to properly control for implementation of some of the identified strategies in the control sub-location due to the nearness in terms of distance of the control sub-location to the experimental sub-locations. Majority of the residents from Central (control sub-location) regularly move to the experimental sub-locations since the experimental sub-locations hosts the central business area, the District Hospital, the bus station and the largest market in Malindi Town. The nearness means that some spill-over effects such as of the town announcements, the posters, banners and leaflets were experienced by the populations of the control sub-location. Another challenge was the use of radio message to create awareness about the MDA. This was not possible as it would have been difficult to make announcements and expect the radio station to isolate it to cover only the experimental sub-locations again due to the nearness of the study sub-locations.

## Discussion

The results reported in this paper indicate that not being aware of the MDA was one of the reasons for not receiving drugs and thus low treatment coverage in Malindi Urban area. Notably, the results of the study have also shown that treatment coverage was lower among the high socio-economic strata and are similar to those of a study conducted in urban areas of Pondicherry, Southern India [25]. Aswathy et *al*., [26] on perceptions and practices of MDA against filariasis in a rural community also showed that a large proportion of the people did not know the term ‘mass drug administration’ although they lived in an area that had experienced three rounds of MDA in their lifetime. This suggests a need for adequate awareness creation, health education and involvement of the target audience in deciding on the materials and methods to be used. Perception of being at risk of infection among high socio-economic groups has also been found to be low in several studies including in Southwestern Ethiopia [27] where health education activities were very weak and lacked epidemiological information that could have probably raised perceived risk of individuals to the disease.

The CDDs not visiting the households to issue the drugs was another reason for low treatment coverage in the urban area. Several reasons are attributable to the CDDs failure to visit the households to issue the drugs including inadequate number of CDDs to cover a large number of households in a limited distribution period. A study on factors associated with CDDs' motivation in rural Kenya, [21] similarly reported that the CDDs themselves viewed their number and length of the distribution period as inadequate for them to effectively conduct the exercise and to interact well with the community members.

Results of the current study also showed that the sources of MDA information influenced treatment coverage. The CDDs followed by other villagers were the most common sources of MDA information across the three socio-economic strata, while the hospital and radio were less common sources of information in the three areas indicating possible lack of reliability of information from CDDs by majority of those in the high socio-economic strata. Similarly, a study on social mobilization and compliance with MDA in rural Kenya [28] revealed that the health professionals did not play a frontline role in disseminating information on MDA suggesting that the information received by some of the community members may have been inadequate and/or incorrect. CDDs and other villagers are non- health professionals who should not be entirely relied upon to disseminate the information.

The current study results indicated that low proportions of household heads in the three socio-economic strata indicated that they felt that the type of information they received about MDA was reliable. Amarillo et *al*., [29] mentioned the important role of the health workers as the community's major source of information indicating that their active and sustained participation is vital in running a five- year MDA programme to eliminate LF. Findings of the results of the study by Njomo *et al*., [21] showed that the CDDs themselves felt that there was inadequacy in source, content and frequency of informing the communities about MDA and called for a combined effort by health workers, local administration and mass media for improved treatment coverage.

Results of the current study also showed that although low proportions of the populations in the three socio-economic strata were of the opinion that the drugs should be distributed using house-to-house method, high proportions in the three socio-economic strata still preferred the house-to-house method in subsequent MDA rounds. Reasons given for this preference were similar to those of a study conducted in Colombo, an urban area in Sri Lanka which sighted reaching many people for treatment [17].The ability of the household heads to give verbal consent for members of his/her household to take the drugs has been indicated to possibly be an important factor for a preference of the house-to-house method of drug distribution [30]. Furthermore, the results similar to those of a study conducted in Bijapur district, India [31] also indicated the importance of revisiting households where members are missed on initial visits in order to reach those who are out at work and increase coverage. Similarly in India, Ranganath and Reddy [32] have highlighted the importance of revisits by the drug distributors. The study results also pointed towards the need of increasing the number of CDDs and the drug distribution period as well as the importance of issuing the CDDs with identification badges and T-shirts so as to give them a sense of ownership of the programme and make them more acceptable to the community members which is similar to the results of the study by Njomo et.*al* [21].

Finally, based on the discussion of the current study and that of other studies, for increased treatment coverage there is a need to educate and mobilize the community members on all aspects of the Programme well in advance. The health personnel should take a lead role in educating the communities. All leaders and stakeholders need to be involved in making the community members understand the benefits of taking the drugs. The drug distributors need to be adequately trained, remunerated and their numbers increased for improved interaction and treatment coverage.

## Conclusion

This study presents the important role played by various community sensitization methods using various materials for increased treatment coverage and successful MDA campaigns in urban areas. First, it is important to involve all stakeholders and community representatives in the MDA planning and implementation process. Importantly, the Programme Implementers should involve all leaders in community mobilization for better awareness creation about MDA and its benefits. Secondly, the numbers of CDDs as well as the length of distribution period need to be increased for improved interaction with the community members. The CDDs need to be provided with identification badges and adequate remuneration due to high standard of living in urban areas. Thirdly, the house-to-house method of distribution is important for acquiring consent from urban household heads and consequently achievement of high treatment coverage.

## References

[1] World Health Organization (1995b) World Health Report, Geneva. pp118.

[2] World Health Organization (2000c) Operational guidelines for rapid mapping of Bancroftian filariasis in Africa World Health Organization.

[3] World Health Organization (2004) Lymphatic filariasis: progress of disability prevention activities. Wkly Epidemiol Rec 79: 417–24. 15595525

[4] Bockarie M, Molyneux D (2009) The end of lymphatic filariasis? Tropical Diseases. British Medical Journal 338: b 1686.

[5] Micheal E, Bundy DA, Grenfell BT (1996) Re-assessing the global prevalence and distribution of lymphatic filariasis. Parasitology 112: 409–428. 8935952 10.1017/s0031182000066646

[6] Njenga SM, Wamae CN, Njomo DW, Mwandawiro CS, Molyneux DH (2008) Impact of two rounds of mass treatment with diethylcarbamazine plus albendazole on *Wuchereriabancrofti* infection and the sensitivity of immunochromatographic test in Malindi, Kenya. Trans R Soc Trop Med Hyg 102: 1017–1024. 18550135 10.1016/j.trstmh.2008.04.039

[7] Centres for Disease Control and Prevention (1993) Recommendations of the International Task Force for Disease Eradication. MMWR Recomm Rep 42: 1–38. 8145708

[8] Moulia-Pelat JP, Glaziou P, Weil GJ, Nguyen LN, Gaxotte P, et al (1995) Combination ivermectin plus diethylcarbamazine, a new effective tool for control of lymphatic filariasis. Trop Med Parasitol 46: 9–12. 7631132

[9] Ottesen EA, Ramachandran C (1995) Lymphatic filariasis infection and disease: Control Strategies. Parasitology Today 11: 129–131.

[10] Ismail MM, Jayakody RL, Weil GJ, Nirmalan N, Jayasinghe KS, et al (1998) Efficacy of single dose combination of albendazole, ivermectin and diethylcarbamazine for the treatment of bancroftian filariasis. Trans R Soc Trop Med Hyg 92: 94–97. 9692166 10.1016/s0035-9203(98)90972-5

[11] Ottesen EA (2000) The global programme to eliminate lymphatic filariasis. Trop Med Int Health 5: 591–594. 11044272 10.1046/j.1365-3156.2000.00620.x

[12] Lammie P, Milner T, Houston R (2007) Unfulfilled potential, using diethylcarbamazine- fortified salt to eliminate lymphatic filariasis. Bull World Health Organ 85: 545–9. 17768503 10.2471/BLT.06.034108 PMC2636360

[13] Stolk WA, Swaminathan S, van Oortmarssen GJ, Das PK, Habbema JD (2003) Prospects for elimination of bancroftian filariasis by mass drug treatment in Pondicherry, India: a simulation study. J Infect Dis 188: 1371–1381. 14593597 10.1086/378354

[14] Plaisier AP, Subramanian S, Das PK, Souza W, Lapa T, et al (1998) The LYMFASIM simulation program for modeling lymphatic filariasis and its control. Methods Inf Med 37: 97–108. 9550853

[15] Ichimori K, Ottesen EA (2011) Eliminating Lymphatic Filariasis. Bol Med Hosp Infant Mex 68 (2) 1–6.

[16] World Health Organization (2008) Global Programme to eliminate lymphatic filariasis: progress report on mass drug administration. Wkly Epidemiol Rec 83: 333–348.18788146

[17] Weerasooriya MV, Yahathugoda CT, Wickramasinghe D, Gunawardena KN, Dharmadasa RA, et al (2007) Social mobilization, drug coverage and compliance and adverse reactions in a Mass Drug Administration (MDA) Programme for the Elimination of Lymphatic Filariasis in Sri Lanka. Filaria J 6: 6–11. 18005398 10.1186/1475-2883-6-11 PMC2203982

[18] Babu BV, Kar SK (2004) Coverage, compliance and some operational issues of mass drug administration during the programme to eliminate lymphatic filariasis in Orissa India. Trop Med Int Health 9: 702–709. 15189460 10.1111/j.1365-3156.2004.01247.x

[19] World Health Organization (2004) Lymphatic Filariasis Elimination Programme. Training module for drug distributors in countries where lymphatic filariasis is not co-endemic with onchocerciasis.

[20] Njomo DW, Amuyunzu-Nyamongo M, Mukoko DA, Magambo JK, Njenga SM (2012) Socio-economic factors associated with Compliance with Mass Drug Administration for Lymphatic Filariasis Elimination in Kenya: Descriptive study results. Ann Trop Med Public Health 5: 103–10.

[21] Njomo DW, Amuyunzu-Nyamongo M, Magambo JK, Ngure PK, Njenga SM (2012) Factors associated with the motivation of community drug distributors in the Lymphatic Filariasis Elimination Programme in Kenya. South Afr J Epidemiol Infect 2: 66–70.

[22] Kenya Population and housing census highlights (2009) Kenya National Bureau of Statistics. pp. 1–8.

[23] Carter A (2010) Factors that contribute to the low uptake of skilled care during delivery in Malindi, Kenya. SIT Study Abroad

[24] Khan ME, Anker M, Patel BC, Barge S, Sadhwani H, et al (1991) The use of focus groups in social and behavioral research: some methodological issues. World HealthStatistics Quarterly 44: 145–149. 1949882

[25] Nandha B, Sadanandane C, Jambulingam P, Das PK (2007) Delivery strategy of mass annual single dose DEC administration to eliminate lymphatic filariasis in the urban areas of Pondicherry, South India: 5 years of experience. Filaria Journal 6: 7 doi:10.1186/1475-2883-6-7 17718908 10.1186/1475-2883-6-7 PMC2020462

[26] Aswathy S, Beteena K, Leelamoni K (2009) Mass drug administration against filariasis in India: perceptions and practices in a rural community in Kerala. Annals ofTropical Medicine & Parasitology Vol. 103 7: 617–624. 10.1179/000349809X12459740922255 19825283

[27] Yirga D, Deribe K, Woldemicheal K, Wendafrash M, Kassahun W (2010) Factors associated with compliance with community directed treatment with ivermectin for onchocerciasis control in Southwestern Ethiopia. Parasites and Vectors 3: 3–48. 20525182 10.1186/1756-3305-3-48 PMC2896929

[28] Njomo DW, Amuyunzu-Nyamongo M, Mukoko DA, Magambo JK, Njenga SM (2012) Social Mobilization and Compliance with Mass Treatment for Lymphatic Filariasis Elimination in Kenya. Afr J Health Sci 20: 42–49.

[29] Amarillo ML, Belizario VY Jr, Sadiang-abay JT, Sison SA, Dayag AM (2008) Factors associated with acceptance of mass drug administration for the elimination of lymphatic filariasis in Agusan del Sur, Philippines. Parasites and Vectors 1: 1–14. 18505577 10.1186/1756-3305-1-14 PMC2441609

[30] Kyelem D, Biswass G, Bockarie MJ, Bradley MH, El-Setouhy M, et al (2008) Determinants of Success in National Programs to Eliminate Lymphatic Filariasis: A Perspective Identifying Essential Elements and Research Needs. Amer Journal Trop MedHyg 79 (4) 480–484. PMC2694403 18840733

[31] Muralidhar MK, Veena GK, Sujatha K, Darshan BB, Varun NA (2013) Coverage and compliance of mass drug administration programme against filariasis in Bijapur District, Karnataka. J Pub Health Med Res 1 (1) 1–4.

[32] Ranganath TS, Reddy NR (2012) Elimination of Lymphatic Filariasis, Mass Drug Administration in Endemic Areas of (Bidar District) Karnataka. Indian J Community Med 37: 219–22. 23293434 10.4103/0970-0218.103468 PMC3531013

